# Distribution of malaria vectors and incidence of vivax malaria at Korean army installations near the demilitarized zone, Republic of Korea

**DOI:** 10.1186/s12936-016-1301-y

**Published:** 2016-05-05

**Authors:** Kyu Sik Chang, Dae-Hyun Yoo, Young Ran Ju, Wook Gyo Lee, Jong Yul Roh, Heung-Chul Kim, Terry A. Klein, E-Hyun Shin

**Affiliations:** National Institute of Health, Korea Center for Disease Control and Prevention, Cheongju, Chungbuk Province 28159 Republic of Korea; 5th Medical Detachment, 168th Multifunctional Medical Battalion, 65th Medical Brigade, Unit 15247, Apo, AP 96205-5247 USA; Public Health Command District-Korea (Provisional), 65th Medical Brigade, Unit 15281, Apo, AP 96205-5281 USA

**Keywords:** Malaria, *Anopheles*, *Plasmodium vivax*, Demilitarized zone, Korea Army-installation

## Abstract

**Background:**

As a result of the reintroduction of malaria in the Republic of Korea (ROK) in 1993 and the threat to military and civilian populations, the Korea Military National Defense (MND) increased emphasis on vector control in 2012 at ROK Army (ROKA) installations located near the DMZ, while decreasing chemoprophylaxis, fearing potential drug resistance. Mosquito surveillance demonstrated a need for continuous monitoring of disease patterns among ROKA soldiers and vector malaria infection rates to ensure positive outcomes.

**Methods:**

*Anopheles* spp. were collected from May–October at three ROKA installations in three locations near the DMZ. Each of the areas included one installation <2 km and two installations 11–12 km from the DMZ in Paju and Yeoncheon counties, Gyeonggi Province. *Anopheles* spp. were identified by polymerase chain reaction (PCR) techniques and then assayed for the presence of vivax malaria sporozoites. The ROK MND reported vivax malaria patients monthly to Korea Centers for Disease Control and Prevention. Correlations for the incidence of *Plasmodium vivax* patients and infected *Anopheles* species were analysed using the Wilcoxon rank sum test, Pearson correlation test and liner regression analysis.

**Results:**

A total of 4282 *Anopheles* spp. were collected. *Anopheles kleini* (69.5 %) was the most commonly collected, followed by *Anopheles pullus* (17.3 %), *Anopheles belenrae* (4.5 %), *Anopheles sineroides* (4.2 %), *Anopheles sinensis* sensu stricto (2.7 %), and *Anopheles lesteri* (1.9 %). Overall, 21 malaria patients were reported by the ROK MND. The monthly incidence of the malaria patients correlated with the monthly number of *Plasmodium vivax* sporozoite positive *Anopheles* spp. The monthly numbers of *An.kleini* demonstrated the highest correlations to the numbers of ROKA malaria patients throughout the mosquito season (P < 0.01). *Anopheles* spp. positive for *P. vivax* and malaria patients at ROKA installations located <2 km from the DMZ were higher than for ROKA installations located 11–12 km from DMZ.

**Conclusion:**

The number of *Anopheles* spp. positive for *P. vivax* sporozoites correlated with the monthly number of malaria cases and exposure of ROKA soldiers from May–October to *P. vivax* malaria infections. Malaria vector surveillance and vector control is warranted as part of an effective malaria management program at ROKA installations located near DMZ.

## Background

The Republic of Korea (ROK-South Korea) was declared malaria free in 1979 by the World Health Organization (WHO) [1]. However, in 1993 a ROK Army (ROKA) soldier stationed near the demilitarized zone (DMZ), a 4-Km wide area separating South and North Korea, was diagnosed with vivax malaria and had no prior travel history to malaria endemic areas outside of the ROK [2, 3]. The number of annual malaria cases (excluding imported cases) increased annually until peaking at 4142 cases in 2000. The number of vivax malaria cases remained over 1000 through 2010, except for 2004 (826), before decreasing below 1000 annual cases in 2011 (762) through 2013 (385) [4]. Vivax malaria cases among ROKA soldiers, including veterans that were discharged from the ROKA <2 years, decreased from 90.5 % (1994) to 57.1 % (2001) and remained below 50.0 % from 2001 to 2015, except for 2009 (51.6 %) and 2011 (57.6 %) [4, 5].

Although *Anopheles* spp. are distributed throughout the ROK, the majority of vivax malaria cases have been attributed to exposure near the DMZ in northern Gyeonggi and Gangwon provinces [6–10]. The WHO reported that malaria transmission near the DMZ may be partially attributed to high numbers of malaria cases in the Democratic People’s Republic of Korea (DPRK) and ecological nature of the DMZ, e.g., unmanaged low-lying areas (abandoned rice paddies following the Korea war) in the DMZ and the exposure of large numbers of ROKA soldiers to malaria vectors [3, 5, 8–12]. Malaria vector control at ROKA installations near the DMZ has been limited as a result of their proximity and sensitivity of military activities near the DMZ that borders the DPRK. *Anopheles* spp. are most active from 22:00 to 02:00 [13, 14], but adult control using hot and ultra-low volume fogging were banned by the ROKA during the evening hours for security reasons. Thus, to reduce the impact of malaria transmission among ROKA soldiers and local civilian populations, malaria management has depended largely on mass chemoprophylaxis (hydroxychloroquine weekly and terminal prophylaxis with primaquine for 14 days) in malaria high-risk (>100 malaria cases/1000 civilians) areas near the DMZ. Additionally, only limited monitoring of malaria vectors has been conducted at ROKA installations located near the DMZ, in part due to limited manpower and costs associated with the identification of members of the *Anopheles* Hyrcanus Group, *Anophelessinensis s.s*., *Anopheles sineroides*, *Anopheles lesteri*, *Anophelespullus*, *Anopheles belenrae*, and *Anopheles kleini*, by polymerase chain reaction (PCR) since they cannot be reliably identified by morphological methods [15, 16]. Therefore, a preliminary investigation to identify seasonal population distributions and vivax malaria sporozoite rates of *Anopheles* spp. was conducted at nine ROKA installations located near the DMZ.

## Methods

### Mosquito collection sites

**Fig. 1:**
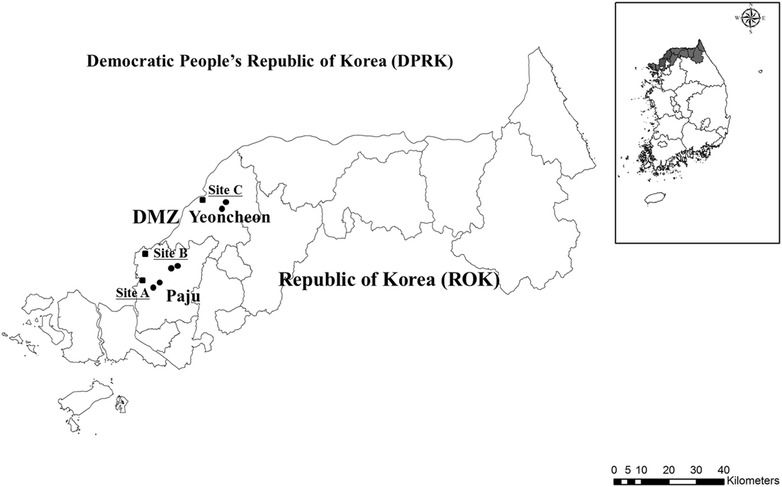
Nine ROKA installations surveyed in Paju and Yeoncheon counties, Gyeonggi Province, Republic of Korea. The* squares* (located <2 km from the DMZ) and* circles* (located 11–12 km from the DMZ) denote the general location of the ROKA installations that were surveyed

The DMZ is a 248 km long and 4 km wide, mined, and heavily fortified zone that divides the Korean peninsula across the 38th parallel and serves as an armistice buffer zone between the ROK and the DPRK. The DMZ consists of forested hills and low-lying unmanaged grasslands (abandoned rice paddies since the end of the Korean War in 1953) that flood during rains and serve as mosquito breeding sites [5]. A total of nine ROKA installations were surveyed, of which three each were located in central Paju (A), northern Paju (B), and Central Yeoncheon (C) counties (Fig. 1). One installation for each of the primary areas surveyed was located <2 km from the DMZ, while the other two installations for each of the areas were located 11–12 km from the DMZ. ROKA installations <2 km from the DMZ housed approximately 500 ROKA soldiers and were bordered by nearby small villages, forested hillsides, and adjacent farmland, consisting of mostly wetland rice farming that serves as mosquito breeding sites. Each of the ROKA installations located 11–12 km from the DMZ housed approximately 700 soldiers and were bordered by small to large villages, forested hillsides, wetland rice farming, and other agriculture. Soldiers from the six installations that were 11–12 km from the DMZ were placed on 6-month rotations to installations located <2 km the DMZ.

### Mosquito collections

Adult mosquitoes were collected weekly using black light traps (Yoshizawa type, black light FL-6w, Shinyoung Co., Seoul) from May–October 2011 at the nine ROKA installations located in malaria high-risk (>1000 malaria cases/100,000 civilians) areas near the DMZ in Paju and Yeoncheon counties, Gyeonggi Province (Fig. 1). Black light traps were set 1.5 m above the ground near barracks, guard posts, and dining facilities and operated for two continuous trap nights/week from 18:00 to 07:00. Mosquitoes were collected after each trap night and trap contents transported to the 5th Medical Detachment, Yongsan US Army Garrison, Seoul, where they were identified to species (culicines) or genus (*Anopheles* Hyrcanus Group). *Anopheles* spp. were placed individually in 2 ml cryovials containing 100 % ethanol and provided to the Korea Centers for Disease Control and Prevention (KCDC) for species identification by PCR.

#### *Plasmodium vivax* malaria patients

The ROK Ministry of National Defense (MND) reported the numbers of vivax malaria cases monthly to KCDC and the monthly numbers of vivax malaria cases tabulated for each of the nine ROKA installations surveyed [13, 14]. The period from the onset of symptoms to diagnosis and treatment were not reported.

### Mosquito identification and *Plasmodium vivax* detection

*Anopheles* spp. were identified to *Anopheles* Hyrcanus Group using morphological keys [17–19]. Species that were only identified to *An.* Hyrcanus Group were identified to species by PCR using genomic DNA extracted from single legs of individual adult mosquitoes as described by Wilkerson et al. and Li et al. [15, 20]. PCR products were separated on 2 % agarose gel and visualized with Safe-Pinky DNA Gel staining solution (×10,000) (GenDepot, TX, USA). Fragment sizes were estimated using molecular weight standards provided by 100-bp ladder molecular weight DNA marker (Bioneer, Seoul, ROK). After mosquitoes were identified to species, the anterior (head and thorax) of each mosquito was separated and assayed for genomic *P*. *vivax* DNA using single step and semi-nested multiplex-PCR as described by Li et al. [15]. Detection and identification of malaria species were simultaneously performed by sequencing two (semi-nested) PCR products [21] and the sizes of the products estimated after electrophoresis on 2 % agarose gels and staining with Safe-Pinky DNA Gel staining solution (×10,000). A Custom AccuPower^®^ Hotstart PCR PreMix (100 mM Tris, 15 mM Mgcl_2_, 400 Mm KCl, 1U Top polymerase, 1U PPase, stabilizer and 0.025 dye) (Bioneer, Daejeon, ROK) was used for the detection of vivax malaria sporozoites in the head and thorax of individual mosquitoes. Known positive and negative samples from previous *P. vivax* positive and negative specimens were used as controls. Genomic DNA of *P*. *vivax* from blood of malaria patients was used as known positive samples. Fragment sizes were estimated by comparison to molecular weight standards provided by 100-bp Ladder Molecular Weight DNA Marker (Bioneer, Seoul, ROK) and genomic DNA fragments sequenced to confirm *Anopheles* spp. positive for *P. vivax* sporozoites [22]. Accession numbers (KU569496, KU569497 and KU569498) for sequences of the 18s RNA gene fragments of *P*. *vivax* from *An*. *kleini*, *An*. *lesteri*, and *An*. *pullus*, respectively are available at the National Center for Biotechnology.

### Data analysis

The minimum field infection rates (MFIR) [(number of pools of *Anopheles* spp. positive for *P. vivax*/number of female mosquitoes) × 100] for flavivirus infections in mosquitoes was used as it estimates the infection rates itself [23, 24]. The correlation of the number of *Anopheles* mosquitoes positive for *P. vivax* sporozoites and malaria patient occurrence was analysed using the Pearson Correlation Test (SAS DOC, version 9.3, SAS Institute, Cary, NC, 2010) [25]. The distributions of *Anopheles* spp. positive for *P. vivax* sporozoites and vivax malaria cases at ROKA installations <2 km and 11–12 km from the DMZ were statistically analysed using the Wilcoxon rank sum test [25]. Linear regression analysis was used for the development of the model for estimating the occurrence of vivax malaria patients based on the MFIR of *Anopheles* mosquitoes positive for *P. vivax* sporozoites.

## Results

### *Anopheles* mosquito species and *Plasmodiumvivax* cases

A total of 7580 female *Anopheles* spp. (4282; 56.5 %) and culicine mosquitoes (3298; 43.5 %), comprised of six genera and 21 species, were collected at nine ROKA installations from May–October (Table 1). *Anopheleskleini* (69.5 %) was the most frequently collected *Anopheles* spp., followed by *An*. *pullus* (17.3 %), *An*. *belenrae* (4.5 %), *An*. *sineroides* (4.2 %), *An*. *sinensis* (2.7 %) and *An*. *lesteri* (1.9 %). *Anopheleskoreicus* and *Anopheleslindesayi japonicus* were not collected. A total of 244/4282 (5.7 %) *Anopheles* mosquitoes from the nine ROKA installations were positive for *P*. *vivax* sporozoites. *Anopheleskleini* was the most frequently collected *Anopheles* spp. positive for *P. vivax* sporozoites (163; MFIR, 5.5 %), followed by *An*. *pullus* (44; 6.0 %), *An*. *sinensis* (13; 11.4 %), *An*. *belenrae* (12; 6.3 %), *An*. *sineroides* (6; 3.3 %) and *An*. *lesteri* (6; 7.3 %) (Table 1).

**Table 1:** Total number (percent) of female *Anopheles* mosquitoes collected, by species, and number positive for vivax malaria by PCR

Species^a^	No. females^b^ (%)^c^	Number positive for vivax sporozoites (MFIR)^d^
*Anopheles kleini*	2975 (69.5)	163 (5.5)
*A. pullus*	739 (17.3)	44 (6.0)
*A. belenrae*	192 (4.5)	12 (6.3)
*A. sineroides*	180 (4.2)	6 (3.3)
*A. sinensis*	114 (2.7)	13 (11.4)
*A. lesteri*	82 (1.9)	6 (7.3)
Total	4282	244 (5.7)

^a^Mosquitoes were collected by black-light traps at 9 ROKA installations <2 km (3) and 11–12 km (6) from the DMZ, Paju and Yeoncheon counties, Gyeonggi Province, Republic of Korea, from May–October, 2011

^b^Total number of female *Anopheles* mosquitoes collected at nine ROKA installations

^c^Each species/total collected

^d^ *MFIR* minimum field infection rates; number positive for vivax malaria sporozoites/total number collected, by species

**Fig. 2:**
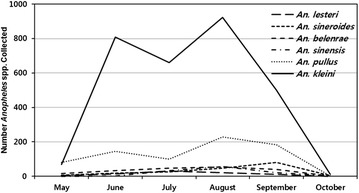
Number of *Anopheles* spp. collected monthly, by species, at 9 ROKA installations located <2 km from the demilitarized zone (DMZ) (*three* installations) and 11–12 km from DMZ (*six* installations)

While the seasonal trap rates for each of the *Anopheles* spp. was variable, they followed similar seasonal patterns (Table 2; Fig. 2). *Anopheles belenrae*, *An*. *pullus*, *An*. *sineroides*, and *An*. *kleini* were collected from May–October, while *An. lesteri* and *An. sinensis* were collected only from May–September and June-October, respectively (Fig. 2). Trap rates for *An*. *kleini* (12.8), *An*. *pullus* (3.2), *An*. *sinensis* (0.8) and *An*. *belenrae* (0.8) were highest during August, while trap rates for *An*. *lesteri* (0.4) and *An*. *sineroides* (1.1) were highest during July and September, respectively.

**Table 2:** The number female *Anopheles* spp. positive for *P. vivax* sporozoites and total number collected, by species, and seasonal correlations between vivax malaria positive *Anopheles* species and vivax malaria cases among ROKA soldiers at the nine ROKA installations located <2 km and 11–12 km from the DMZ, Paju and Yeoncheon counties, Gyeonggi Province, ROK, from May–October, 2011

Species	Number of female *Anopheles* spp. positive for *P. vivax* sporozoites/total number collected (MFIR)^a^						Correlation^c^		
	May (0)^b^	Jun (2)	Jul (11)	Aug (6)	Sep (1)	Oct (1)	n	Sig.	Value
*Anopheles lesteri*	1/5 (20.0)	0/13 (0.0)	3/31 (9.7)	1/22 (4.5)	1/11 (9.1)	0/0 (0.0)	6	0.046^d^	0.82
*An. sineroides*	0/7 (0.0)	0/18 (0.0)	1/25 (4.0)	3/46 (6.5)	2/81 (2.5)	0/3 (0.0)	6	0.466	0.374
*An. belenrae*	2/17 (11.8)	1/32 (3.1)	3/46 (6.5)	3/54 (5.6)	3/41 (7.3)	0/2 (0.0)	6	0.287	0.523
*An. sinensis*	0/0 (0.0)	0/1 (0.0)	4/34 (11.8)	5/56 (8.9)	4/20 (20.0)	0/3 (0.0)	6	0.171	0.64
*An. pullus*	6/81 (7.4)	7/146 (4.8)	9/99 (9.1)	14/228 (6.1)	8/184 (4.3)	0/1 (0.0)	6	0.279	0.53
*An. kleini*	8/69 (11.6)	32/809 (4.0)	66/661 (10.0)	48/924 (5.2)	9/501 (1.8)	0/11 (0.0)	6	0.006^e^	0.936
Total	17/179 (9.5)	40/1019 (3.9)	86/896 (9.6)	74/1330 (5.6)	27/838 (3.2)	0/20 (0.0)	6	0.012^d^	0.911

^a^Number of vivax malaria positive *Anopheles* sp./total number of *Anopheles* spp., by species and month of collection

^b^Percent of *Anopheles* spp. positive for *P. vivax* sporozoites

^c^Correlation between the number of *Anopheles* spp. positive for *P. vivax* sporozoites and vivax malaria patients from the 9 ROKA installations from May–October

^d, e^Correlations significant at P < 0.05 and P < 0.01 by Pearson correlation test, respectively

**Fig. 3:**
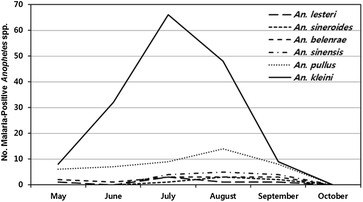
Number of vivax malaria positive *Anopheles* spp. collected monthly, by species, at 9 ROKA installations located <2 km (*three* installations) and 11–12 km (*six* installations) from the DMZ

All *Anopheles* spp. positive for *P. vivax* sporozoites demonstrated variable seasonal distributions. *Anopheleslesteri*, *An*. *belenrae*, *An*. *pullus*, and *An*. *kleini* that were positive for *P*. *vivax* sporozoites were observed during May when mosquito surveillance was initiated, while vivax malaria positive *An*. *sineroides* and *An*. *sinensis* were first observed during July (Table 2; Fig. 3). The numbers of *P*. *vivax*-infected *An*. *kleini* (66; MFIR 10.0 %) and *An*. *lesteri* (3; 9.7 %) peaked during July, while the numbers of *An*. *pullus* (14; 6.1 %), *An*. *sinensis* (5; 8.9 %), and *An*. *sineroides* (3; 6.5 %) peaked in August.

**Fig. 4:**
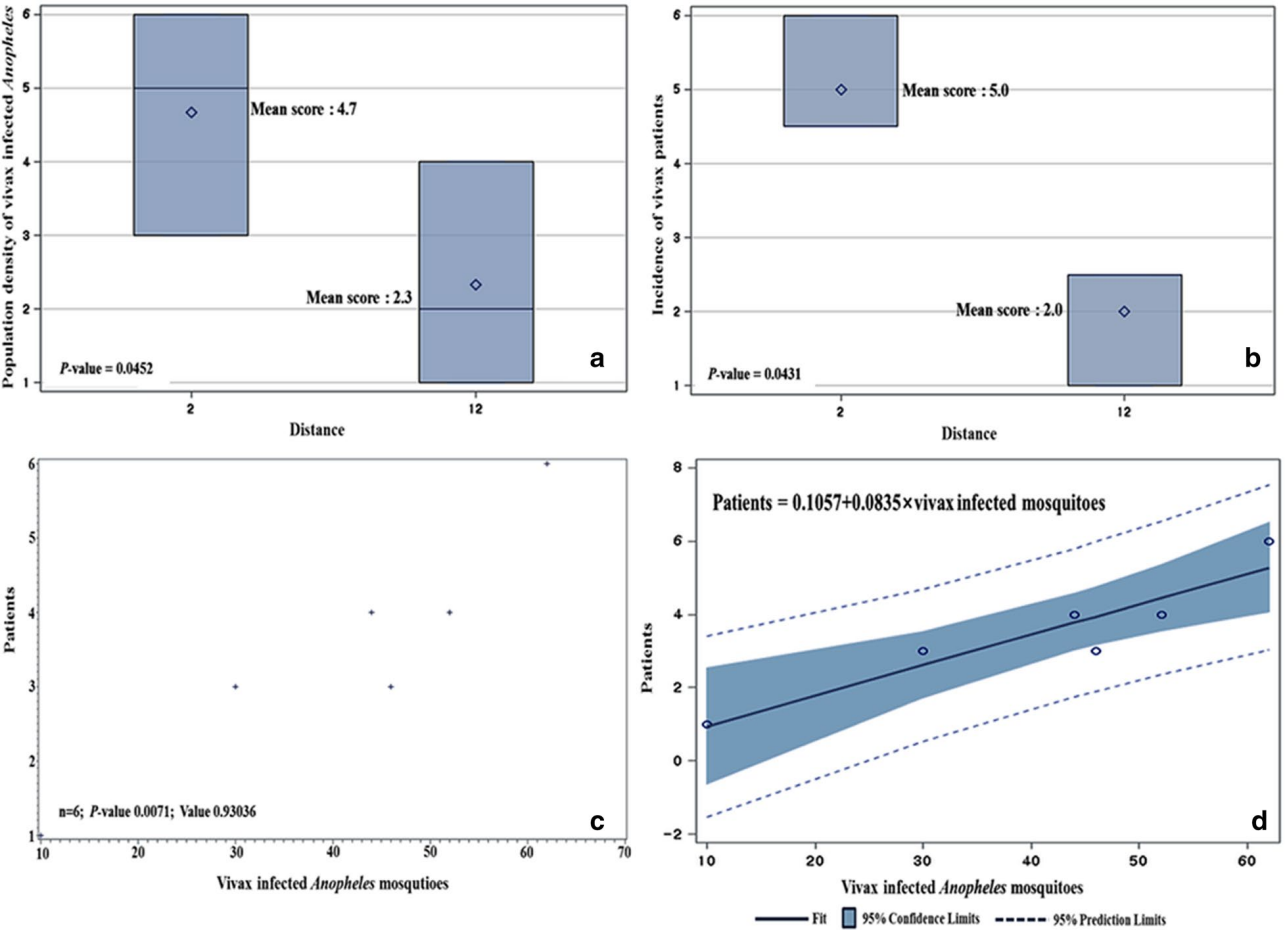
Comparative analysis of vivax malaria patients based on the number of *Anopheles* spp. positive for *P. vivax* sporozoites at 9 ROKA installations located <2 km (*three* installations) and 11–12 km (*six* installations) from the DMZ. **a** Wilcoxon mean scores by Wilcoxon rank sum test for the number of *Anopheles* spp. positive for *P. vivax* sporozoites based on distance (<2 km and 11–12 km) from the DMZ. **b** Wilcoxon mean scores by Wilcoxon rank sum test for number of vivax malaria patients based on distance (<2 km and 11–12 km) from the DMZ. **c** *Scatter plots* by Pearson correlation test based on the number of *Anopheles* spp. positive for *P. vivax* sporozoites and vivax malaria patients at 9 ROKA installations. **d** Model by liner regression analysis to estimate the incidence of vivax malaria positive patients based on number of *Anopheles* spp. positive for *P. vivax* sporozoites

Overall, the seasonal distribution of the *Anopheles* spp. positive for *P. vivax* sporozoites demonstrated similar patterns to seasonal occurrence to vivax malaria cases among ROKA soldiers stationed at the nine surveyed installations (Table 3; Fig. 4). While there were *Anopheles* mosquitoes positive for malaria during May, vivax malaria cases at the ROKA installations were not observed until June (2 cases). The number of vivax malaria cases increased to a high in July (11), then decreased in August (6), with no malaria cases reported during September–October. As a result of low numbers of *Anopheles* mosquitoes collected when trapping was initiated in May, only 17 (MFIR 9.5 %) were positive for *P. vivax* (Table 3). The number of *P. vivax* positive mosquitoes were much higher in June (40; 3.9 %), peaking in July (86; 9.6 %) and then decreasing in August (74; 5.6 %) through September (27; 3.2 %). No *Anopheles* spp. were positive for *P. vivax* in October, which was most likely due to very low numbers collected.

**Table 3:** Seasonal correlation between vivax malaria infected *Anopheles* mosquitoes and vivax malaria cases among ROK Army soldiers stationed at nine ROK Army installations near the DMZ, Paju and Yeoncheon, Gyeonggi Province, Republic of Korea, from May–Oct, 2011

Species	Number of *Anopheles* spp. positive for *P. vivax* sporozoites/total number of collected (MFIR)^a^												Correlation^c^		
	May	MP^b^	Jun	MP	Jul	MP	Aug	MP	Sep	MP	Oct	MP	n	Sig.	Value
Site A	2/44 (4.5)	0	11/475 (2.3)	0	46/472 (9.7)	6	42/631 (7.1)	2	7/218 (3.2)	1	0/8 (0.0)	0	6	0.032^d^	0.851
Site B	4/49 (8.16)	0	21/440 (4.8)	1	38/401 (9.5)	4	15/228 (5.3)	2	4/138 (2.9)	0	0/11 (0.0)	0	6	0.006^e^	0.938
Site C	11/86 (12.8)	0	8/104 (7.7)	1	2/23 (8.7)	1	17/471 (3.6)	2	16/482 (3.3)	0	0/1 (0.0)	1	6	0.943^f^	−0.38
Total	17/179 (9.5)	0	40/1019 (3.9)	2	86/896 (9.6)	11	74/1330 (5.6)	6	27/838 (3.2)	1	0/20 (0.0)	1	6	0.012^d^	0.911

^a^MFIR, Number of *Anopheles* spp. positive for *P. vivax* sporozoites/total number *Anopheles* spp. collected (percent positive)

^b^Number of vivax malaria ROKA soldiers reported from the 9 ROKA installations surveyed

^c^Correlation between the number of *Anopheles* spp. positive for *P. vivax* sporozoites and *vivax* patients reported from the 9 ROKA installations surveyed from May–October, where n = number of months

^d, e^Correlations significant at the P < 0.05 and P < 0.01 levels by Pearson correlation test of SAS, respectively

^f^Not significant

Overall, the numbers of *Anopheles* mosquitoes positive for *P. vivax* showed significant correlations to the number of vivax malaria cases among ROKA soldiers (P < 0.01), but not vivax infected rates of *Anopheles* mosquitoes. However, both *An*. *kleini* and *An*. *lesteri* that were positive for *P. vivax* demonstrated significant seasonal distribution correlations to malaria cases among ROKA soldiers (P < 0.05).

### Surveillance site correlations of seasonal distributions of *Anopheles* mosquitoes positive for *Plasmodium vivax* and ROKA vivax malaria cases

Although correlations that compared the overall rates of *Anopheles* mosquitoes positive for *P. vivax* and vivax malaria cases were not significant, vivax malaria cases among ROKA soldiers and *Anopheles* mosquitoes positive for *P. vivax*, for each of the three areas surveyed [central Paju (A), northern Paju (B), and central Yeoncheon (C)], and for collection sites <2 km and collection sites 11–12 km from the DMZ, demonstrated high correlations in seasonal distributions (P < 0.05) (Table 3). In addition, correlations of *P. vivax* infected *An. kleini* and *An. lesteri*, both implicated as the primary vectors of malaria in the ROK, demonstrated high correlations with vivax malaria cases among ROKA soldiers. The correlation in monthly incidence of *P. vivax* positive *Anopheles* mosquitoes and vivax malaria cases among ROKA soldiers for areas A and B in Paju County, considered to be a malaria high risk area, was high (P < 0.05 and P < 0.01, respectively), but not significant for area C, which may be due, in part, to the low numbers of *Anopheles* spp. collected at one of the installations.

For each of the collection areas A, B, and C (including one collection point <2 km from the DMZ and two collection points 11–12 km from the DMZ), the seasonal distribution of *P. vivax* positive *Anopheles* mosquitoes and vivax malaria cases were monitored (Table 4). *Plasmodium vivax* positive *Anopheles* mosquitoes and vivax malaria cases were higher at all ROKA installations located <2 km compared to those located 11–12 km from the DMZ. Vivax malaria cases for installations <2 km, each housing approximately 500 ROK Army soldiers and located at sites A, B, and C, were 6, 4, and 4, respectively, while installations at sites A, B, and C that were located 11–12 km from the DMZ and housing approximately 1400 (700 each) soldiers were 3, 3, and 1, respectively. A total of 62, 52 and 44 *P. vivax* positive *Anopheles* mosquitoes were observed at ROKA installations <2 km from the DMZ at sites A, B, and C, respectively. A total of 46, 30, and 10 *Anopheles* mosquitoes positive for *P. vivax* sporozoites were observed at both installations (mean 23, 15, and 5) located at sites A, B, and C 11–12 km from the DMZ, respectively.

**Table 4:** Vivax malaria positive *Anopheles* mosquitoes, by species, and total number collected by black-light traps and number of vivax malaria patients at nine ROK Army installations located near the DMZ, Paju and Yeoncheon counties, Gyeonggi Province, ROK, from May–October, 2011

Species	Number of *Anopheles* spp. positive for *P. vivax* sporozoites/total umber (MFIR)^a^						Total positive/(%)
	Site A^b^		Site B^c^		Site C^d^		
	<2 km (6)^e^	11–12 km (3)	<2 km (4)	11–12 km (3)	<2 km (4)	11–12 km (1)	
*Anopheles lesteri*	1/5 (20.0)^e^	0/7 (0.0)	4/56 (7.1)	0/10 (0.0)	1/4 (25.0)	0 (0.0)	6/82 (7.3)
*An. sineroides*	3/72 (4.2)	0/23 (0.0)	1/33 (3.0)	1/31 (3.2)	1/20 (5.0)	0/1 (0.0)	6/180 (3.3)
*An. belenrae*	3/35 (8.6)	0/5 (0.0)	7/115 (6.1)	1/25 (4.0)	1/8 (12.5)	0/4 (0.0)	12/192 (6.3)
*An. sinensis*	0/31 (0.0)	0/29 (0.0)	1/17 (5.9)	8/35 (22.9)	0/0 (0.0)	0/2 (0.0)	13/114 (11.4)
*An. pullus*	10/199 (5.0)	3/56 (5.4)	8/196 (4.1)	12/166 (7.2)	2/54 (3.7)	6/68 (8.8)	44/739 (6.0)
*An. kleini*	45/910 (4.9)	43/476 (9.1)	31/483 (6.4)	8/100 (8.0)	39/957 (4.1)	4/49 (8.2)	163/2975 (5.5)
Total	62/1252 (5.0)	46/596 (7.7)	52/900 (5.8)	30/367 (8.2)	44/1043 (4.2)	10/124 (8.1)	244/4282 (5.7)

^a^Number of vivax malaria patients reported for ROKA installations located <2 km (1 for each area) and 11–12 km (2 for each area)

^b^ROKA installations in central Paju County, Gyeonggi Province

^c^ROKA installations in northern Paju County, Gyeonggi Province

^d^ROKA installations in central Yeoncheon county, Gyeonggi Province

^e^MFIR, Number of *Anopheles* sp. positive for *P. vivax* sporozoites/total number *Anopheles* sp. collected (percent positive)

The distribution of *Anopheles* mosquitoes positive for *P. vivax* sporozoites and vivax malaria patients at ROKA installations <2 km and at 11–12 km from the DMZ were analysed using the Wilcoxon rank sum test (Figs. 2, 3). Wilcoxon mean scores for *P. vivax* positive *Anopheles* mosquitoes at <2 km and 11–12 km from the DMZ was 4.7 and 2.3, respectively (P = 0.0452) and for vivax malaria cases at installations <2 km and 11–12 km from the DMZ were 5.0 and 2.0, respectively (P = 0.0431). A high correlation (P = 0.0071) was observed for the distribution of *Anopheles* mosquitoes positive for *P. vivax* sporozoites and vivax malaria cases based on distances of <2 km and 11–12 km from DMZ using the Pearson method (Fig. 4). A model for the estimation of occurrence of vivax malaria patients based on *P. vivax* positive rates of *Anopheles* mosquitoes is shown in Fig. 4 with linear regression equation of 0.1057 + 0.0835 × *P. vivax* positive *Anopheles* mosquitoes.

## Discussion

Since 2002, the ROKA veteran and active duty soldiers accounted for 28.0–57.6 % of all vivax malaria cases (excluding imported cases) in the ROK (KCDC, 2015) [26]. Nearly all of the malaria cases among ROK military and veterans discharged <2 years after military service were acquired while stationed at ROKA installations and while conducting military training at sites located near the DMZ in northern Gyeonggi and Gangwon Provinces. These data illustrate the requirement for effective malaria control measures to be instituted at ROKA installations and training sites located near the DMZ. The ROK MND has previously relied heavily on chemoprophylaxis for malaria control for areas considered to be malaria high-risk, since vector control measures were difficult to implement due to security and sensitivity of these operations near the DMZ that borders the DPRK. However, due to the fear for the development of drug resistance, in addition to high costs associated with chemoprophylaxis, the Korea MND decreased the use of chemoprophylaxis from 2011 to 2013 [27–29]. To compensate for the reduction in chemoprophylaxis and still reduce malaria risks, the ROK MND increased pesticide use for vector control from 2011 to 2013 when there were corresponding decreased numbers of malaria cases among civilian, military, and veteran populations from 762 to 385 cases. The ROK MND changed its policy in 2014, increasing the use of anti-malarial drugs while decreasing the use of pesticides for vector control, which resulted in similar numbers of malaria cases among ROKA active duty and veteran populations during 2013–2014, while the number of malaria cases among civilians increased by 77.1 %. These results indicate that vector control at ROKA installations impacted positively on civilian populations by reducing malaria rates among the civilian populations. These data further indicate the importance for implementing both chemoprophylaxis and vector control policies at ROKA installations located near the DMZ to not only reduce malaria cases among ROKA soldiers, but to also reduce the impact of malaria infections among civilian populations.

A better understanding of vector geographical and seasonal distributions, anthropophilic behavior, and vector potential is essential for the development of an effective malaria control programme [5]. *Anopheles kleini* was the predominant *Anopheles* spp. collected by black light near human habitation, whereas *An. sinensis* was the predominant *Anopheles* spp. collected by CDC light trap and resting collections at cow sheds (71.7 %), followed by *An. kleini* (19.05) located approximately 3 km from the DMZ [30]. While the differences in the proportion of *An. sinensis* are likely due to greater zoophilic attraction, trapping methods, e.g., black light versus CDC light traps, Mosquito Magnet traps, and resting collections cannot be discounted. Therefore differences in zoophilic and anthropophilic attraction must be investigated to more precisely identify the impact of each *Anopheles* spp. in order to develop an effective targeted vector control program that reduces associated vector and malaria control costs.

**Fig. 5:**
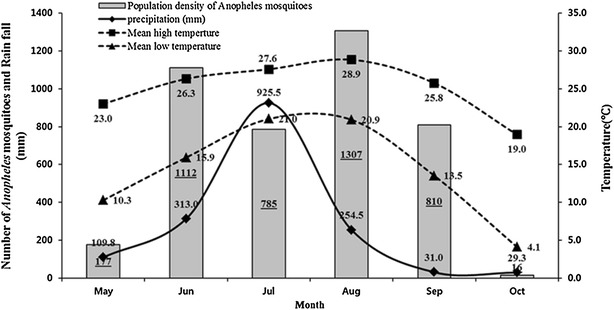
Mean monthly high and low temperatures (^o^C), precipitation^a^, and total number of *Anopheles* mosquitoes collected monthly at black lights from nine ROKA installations from May–October, 2011. ^a^ Available at Korea meteorological administration: http://203.247.66.10/weather/observation/aws_table_popup.jpg

While all six members of the *Anopheles* Hyrcanus Group collected during this survey have been implicated as potential vectors of *P*. *vivax*, their role in malaria transmission is poorly understood [31–36]. Based on both adult and larval collections, *An. kleini* is the predominant mosquito collected during the early summer, while *An. sinensis* is the predominant larvae collected during the fall, which may be due to habitat changes over the mosquito breeding season [37, 38] The reduction in population densities during July likely resulted from heavy monsoon rains (1851.0 mm) that accounted for over 50 % of total rainfall (3477.5 mm) in Paju and Yeoncheon counties during 2011 (Fig. 5). Following the heavy rainfall in July, *Anopheles* populations likely increased in August as a result of warm temperatures and low-lying areas that remained flooded and the continued flooding of rice paddies. In September–October, populations of *Anopheles* mosquitoes sharply decreased as rains subsided, flooded low lying areas dried up, rice paddies were drained for harvesting, and temperatures cooled.

*Anopheles kleini* was the predominant *Anopheles* spp. captured at black light traps from June to October when high numbers of malaria cases were reported, and while the MFIR was only 5.5, the number of specimens positive for *P*. *vivax* was much greater than for all the other species combined and for all collection sites. Preliminary data also suggests that *An*. *kleini* is a primary vector of *P*. *vivax* malaria in the ROK [7, 33, 36]. Additionally, laboratory studies showed high numbers of sporozoites in the salivary glands of *An. kleini* [33], whereas *An. sinensis* demonstrated very low numbers (<10) sporozoites in the salivary glands when provided blood meals on blood obtained from Thai patients positive for *P. vivax* malaria (Ubalee, unpublished data). A study by Golenda et al. showed that *Anopheles stephensi* infected with *Plasmodium falciparum* and *Plasmodium berghei* did not eject sporozoites during salivation when there were <10 sporozoites [39]. In addition they demonstrated that as the gland indices increased, the numbers of sporozoites ejected during salivation increased. These data further implicate *An. kleini* as a primary vector since >100–1000 sporozoites have been observed in their salivary glands following feeding on blood from Korean and Thai patients positive for *P. vivax*. Population densities of *An*. *kleini* also are reported to be much higher for malaria high-risk areas near the DMZ compared with malaria low-risk areas south of Seoul where *An*. *sinensis* accounted for >95 % of *Anopheles* collected [7, 35].

Additionally, collections in northeastern Russia, just above the DPRK, found no *An. sinensis*, while *An. kleini* was collected at a frequency of 66.67–100 % of all *Anopheles* spp. collected, suggesting that the DPRK has high populations of *An. kleini* compared to *An. sinensis* [40, 41]. This may, in part, account for the higher numbers of malaria cases that continue to be reported from the DPRK [11, 12]. However, WHO in both the 2011 and 2015 malaria report continues to indicate that *An. sinensis* is the primary mosquito near the DMZ where malaria is transmitted, even though there is data to refute this supposition [7, 11, 12, 35, 37, 40, 41].

Correlations of the monthly incidence of vivax malaria patients and numbers of *Anopheles* spp. captured at black lights at ROKA installations near the DMZ in Paju and Yeoncheon counties, Gyeonggi Province were not significantly different. However, there was a high correlation of the monthly incidence of vivax malaria patients and numbers of *Anopheles* spp. positive for *P. vivax* sporozoites. Therefore, the numbers of primary vector(s) positive for *P. vivax* was considered the primary factor for the monthly incidence of vivax malaria patients based on distance from DMZ. Of the six *Anopheles* species, *An*. *kleini* showed the highest correlation compared to the monthly number of vivax malaria patients, followed by *An*. *lesteri*. *Anopheleskleini* has been reported as the primary vivax malaria vector and predominant species collected near the DMZ since it was first described [16, 30, 34]. Joshi et al. [32, 35] also reported that *An*. *lesteri* demonstrated the highest susceptibility to *P. vivax* when compared to *An*. *sinensis* and *An*. *pullus*, and based on limited surveys, *An. lesteri* may be the predominant vivax malaria vector for some areas in northwestern Gyeonggi Province.

The number of vivax malaria patients and *P. vivax* positive *Anopheles* mosquitoes showed similar monthly incidence patterns, but varied weekly for each of the months. The mean temperatures during April/May ranged from 16 to 20 °C, with an expected sporogonic development period in the mosquitoes of >20 days. While *Anopheles* mosquitoes positive for *P. vivax* sporozoites were collected during May, ROKA malaria patients were not reported until June, suggesting that transmission occurred in late May/early June. The numbers of malaria patients from January to April were not reported since mosquitoes were not collected and malaria cases during that period would have been due to latent malaria cases (symptoms occurring >6 months after transmission) from the previous mosquito season. Mosquitoes are present in April and after blood feeding on civilian, veteran, and active duty military personnel that became ill with latent malaria from the previous season may develop sporozoites by mid-May. While the number of asymptomatic malaria cases with circulating blood stages has not been determined for civilian, military, and veteran populations, it is believed to be very low based on the proximity of available medical care.

Unique conditions in the vicinity of the DMZ, e.g. (1) unmanaged lands within the DMZ (2) surrounding areas that increase mosquito breeding potential, (3) exposure of large numbers of ROKA soldiers to biting mosquitoes during the evening hours, and (4) proximity to the DPRK where there are much higher numbers of vivax malaria cases, increase the potential for malaria transmission [3, 8, 9, 11, 12]. Mosquito control near the military demarcation line (center of the DMZ) that borders the DPRK is not conducted due to the high level of security and sensitivity. The potential for malaria infected mosquitoes flying from the DPRK where high numbers of malaria cases were reported by WHO [11, 12] increases risks for infecting civilians and military personnel residing, stationed, or conducting military training near the DMZ. Therefore, monitoring the monthly incidence of vivax malaria patients and relative *Anopheles* spp. population densities and *P. vivax* sporozoite infection rates is very important to assess the role of malaria vectors at ROKA installations based on distance from the DMZ.

There is a greater potential for exposure to *Anopheles* mosquitoes positive for *P. vivax* sporozoites among ROKA soldiers from May to September based on the Wilcoxon Rank Sum Test. High malaria sporozoite rates among multiple *Anopheles* spp. that were captured within the perimeter of the ROKA installations suggest that alternative preventative measures (e.g. vector control) effectively reduce malaria risks. These results highlight the importance of vector control near the DMZ to reduce the numbers of sporozoite-positive mosquitoes as part of an overall malaria management programme. Furthermore, KCDC assets, e.g., the Korea National Institute of Health (KNIH) and Quarantine stations, must be augmented, when possible, with U.S. preventive medicine assets, e.g., preventive medicine Medical Detachments, local universities, and the Armed Forces Health Surveillance Branch to provide more timely and accurate analysis of information and effective reduction of malaria cases among Korean and US military and civilians residing or working in/near the DMZ. This data provides risk analyses for the ROK military and civilians near the DMZ, in addition to recommendations for instituting effective vector control and disease reduction strategies.
